# Monkeypox clinical symptoms, pathology, and advances in management and treatment options: an update

**DOI:** 10.1097/JS9.0000000000000091

**Published:** 2023-03-01

**Authors:** Sirwan K. Ahmed, Rabab G.A. El-Kader, Salar O. Abdulqadir, Ardalan J. Abdullah, Nahed A. El-Shall, Deepak Chandran, Abhijit Dey, Talha B. Emran, Kuldeep Dhama

**Affiliations:** a Department of Nursing, University of Raparin; b Department of Emergency, Rania Pediatric and Maternity Teaching Hospital, Rania, Sulaimani; c Department of Emergency Nursing, Haibat Sultan Technical Istitute, Koye, Kurdistan Region, Iraq; d RAK College of Nursing, RAK Medical and Health Sciences University, Ras Al-Khaimah, UAE; e Faculty of Nursing, Mansoura University, Mansoura; f Department of Poultry and Fish Diseases, Faculty of Veterinary Medicine, Alexandria University, Edfina, El-Beheira, Egypt; g Division of Pathology, ICAR-Indian Veterinary Research Institute, Bareilly, Uttar Pradesh; h Department of Life Sciences, Presidency University, Kolkata, West Bengal; i Department of Veterinary Sciences and Animal Husbandry, Amrita School of Agricultural Sciences, Amrita Vishwa Vidyapeetham University, Coimbatore, Tamil Nadu, India; j Department of Pharmacy, Faculty of Allied Health Sciences, Daffodil International University, Dhaka, Bangladesh; k Department of Pharmacy, BGC Trust University Bangladesh, Chittagong, Bangladesh

*Dear Editor*,

Monkeypox (MPX), caused by the monkeypox virus (MPXV), a DNA virus of family *Poxviridae*, genus *Orthopoxvirus*, has now spread to 107 countries and territories, and as of October 6, 2022, nearly 70,420 cases have been reported with 27 deaths^1^. Clinical health care for MPX is similar to smallpox^2^. Two distinct phylogenetic clades of MPXV have been identified through genome sequencing, the Central African (Congo Basin) and West African clade. The genetic differences between the viral genomes of the two clades may provide an explanation for variations in viral clearance and pathogenicity^3^. Typically, the Central African MPXV leads to higher rates of transmission and mortality, and more severe disease^3,4^. correspondence article provides up-to-date information on the clinical features, pathogenesis as well as advances in treatment and management options for MPX.

The incubation period of MPXV is usually 6–13 days but may also range 5–21 days. Symptoms such as fever, headache, chills, fatigue, fainting, swelling of lymph nodes, back pain, and muscle aches appear at the onset of MPX disease^2^. However, within 3 days of the onset of these prodromal symptoms, a maculopapular centrifugal rash develops at the site of the initial infection and rapidly spreads to other parts of the body, in which the hands and fingertips are involved as a feature of the disease in cases of disseminated rash. The lesions usually worsen within 12 days, occurring sequentially from the macular stage to papules, tortoises, pus, crust, and fat before falling off^2^. Sometimes ulcers are also formed on the mucous membrane of the mouth or eyes (enanthem). The clinical features of MPX including different signs and lesions may be somewhat difficult to distinguish from smallpox, other orthopoxviruses viruses (cowpox, camelpox, buffalopox viruses), parapoxvirus infections or (pseudocowpox, and bovine popular stomatitis viruses), and to some extent chickenpox^5^. MPXV causes lymphadenopathy (e.g. in the cervix or inguinal lesion), while chickenpox and smallpox virus usually do not, which is the main difference between MPX and smallpox^2^. It should be noted that in travel-related cases in Western countries, clinical symptoms are usually mild, sometimes with very few lessons. However, studies published in 2022 further revealed that many cases presented spots in the anogenital area^6^. A number of patients in the United States who have recently contracted MPX epidemic have complained of anus pain during the beginning of the illness. Rectal inflammation, rectal bleeding, and the need to defecate (when the rectum is empty) are also experienced by patients just diagnosed with MPX in the United States^6^. When MPX patients develop complications, they can be lethal. Lesions on the skin, for instance, can invite subsequent bacterial infections. In addition to causing severe respiratory distress and pneumonia, this illness can cause ocular infections that can cause vision loss, as well as a loss of appetite, vomiting, diarrhea, and cervical lymphadenopathy that can progress into retropharyngeal abscesses. Increased risk of mortality, encephalitis, and neurological complications such sepsis and septic shock can exaggerate clinical outcomes^7,8^.

Most cases of MPX in humans show mild to moderate symptoms with a self-limiting course of the disease. The route of transmission, host susceptibility and vulnerability, and amount of inoculated virus may vary the severity of the disease^9^, with some invasive forms of exposure causing more severe illness but requiring shorter incubation periods^10^. Health problems such as encephalitis, secondary bacterial skin infections, conjunctivitis, dehydration, ear infections, and respiratory distress are complications of the disease in endemic countries. Furthermore, secondary attacks of MPX often occur, which are less common in patients vaccinated against smallpox^4^.

According to WHO guidelines, patients with suspected or confirmed MPX can be isolated at home until the disease is mild, high risk of complications are not observed, and appropriate infection prevention and control measures can be followed. A patient with mild, uncomplicated MPX during home care should be isolated from other household members and be kept away from common areas of the household (i.e. a separate room or a curtain separated from other household members). Needful care should be taken and adequate health and safety measures should be followed when cleaning waste containing linen or when cleaning the house^11^.

Supportive care for patients with MPX comprises of rehydration to balance fluid losses, hemodynamic balance, oxygen supplementations, symptomatic therapies, and limiting bacterial co-infections complicating skin lesions and eye infections with applying lubricants, and topical antibiotics and possibly antivirals (trifluridine)^12,13^. Antivirals including tecovirimat, brincidofovir, and cidofovir, and vaccinia immune globulin intravenous (VIGIV) administration can be beneficial to treat more serious cases of MPX that need hospitalization, complicated lesions, and when lesions appear near eyes, mouth and genitals, patients are immunocompromised/immunosuppressed, pediatrics, younger children within 8 years of age, pregnant and breast-feeding women^13–15^.

MPX patients should be administered symptomatic treatments such as antipyretics and painkillers, and those who are malnourished need adequate nutrition and hydration. Patients with mild MPX should be advised to be aware of symptoms that require urgent care. All secondary infections and skin rashes caused by the disease should be treated. Antibiotic or prophylactic treatment for patients with MPX is not used in the early stages. However, wounds should be monitored to protect them from secondary bacterial infections (i.e. cellular infections, pus). If the patient is sensitive to an antibiotic, alternative antibiotics should be given, for example in case of a problem with the destruction of normal flora including methicillin-sensitive *Streptococcus pyogenes* and *Staphylococcus aureus* ^11^. Patients who are at high risk of complications such as pregnant women, children, and those who are immunosuppressed and are at risk due to severe symptoms should be admitted to hospitals for treatment and health care, and adopt preventive measures to prevent further transmission of the disease^11^.WHO guidelines emphasize the importance of controlling anxiety and depression in MPX patients and paying special attention to psychological aspects. In the framework of mental health care of infected patients, pay attention to proper sleep, which can be disrupted by the sudden mental stress of infection, because it directly affects the treatment and the immune system of the patients^16–18^. Tecovirimat (TPOXX), an antiviral smallpox drug, is the first pharmaceutical of its kind that has been licensed by the European Medicines Agency (EMA), the Food and Drug Administration (FDA), and Health Canada for the treatment of MPX. Animal studies have demonstrated that this medication is effective against multiple orthopoxviruses, including MPX. There were no concerning adverse effects from the compassionate use of tecovirimat for treating severe cases of vaccinia and cowpox^12,19^. Tecovirimat inhibits MPXV envelope formation by inhibiting the viral protein p37, which is highly conserved across Orthopoxviruses. Oral capsules with an instant release formula need to be taken twice daily for a total of 14 days. On May 19, 2022, the USFDA sanctioned an intravenous formulation. A broader distribution plan for the anti-MPX drug tecovirimat is currently in the works for the Central African Republic, which has experienced a number of recent outbreaks^14^. Other choices for MPX treatment options being explored are cidofovir and brincidofovir, two antiviral drugs used to treat cytomegalovirus and human smallpox illness, respectively^12^. An intravenous infusion of vaccinia-specific immunoglobulin, also known as VIGIV, is used to treat side effects of the vaccinia vaccine. The CDC has given approval for use of VIGIV for its use in the treatment of MPX as part of an enhanced access protocol^8^. The use of antivirals is essential for patients with MPX under randomized clinical trials. To develop effective antivirals, more trials are also required, along with sufficient clinical data, expected results, and evidence-based therapeutic outcomes as soon as possible. If this is not achievable, antiviral drug use should be initiated under expanded access protocols such as monitored emergency use of unregistered interventional investigations.

ACAM2000 (a live, replication-competent vaccinia virus) and JYNNEOS (a live, replication-incompetent vaccinia virus; also known as Imvamune or Imvanex or MVA-BN) are the two smallpox vaccines that are now recommended to be used against MPX. Some people have a very painful and uncomfortable cutaneous reaction at the injection site after receiving ACAM2000, however, this is not the case with JYNNEOS because this reaction is not caused by the unchecked reproduction of the virus. The risk of inadvertent and self-inoculation exists for ACAM2000, but not for JYNNEOS^20^. The WHO has issued a worldwide alert about the ongoing multination MPX outbreaks, urging all countries to evaluate the situation and convene their national immunization technical advisory groups to review the data and make vaccine use recommendations tailored to each country’s specific needs^20^.

An overview on clinical features, complications, and treatment aspects of MPX patients is presented in Figure 1.

**Figure 1:**
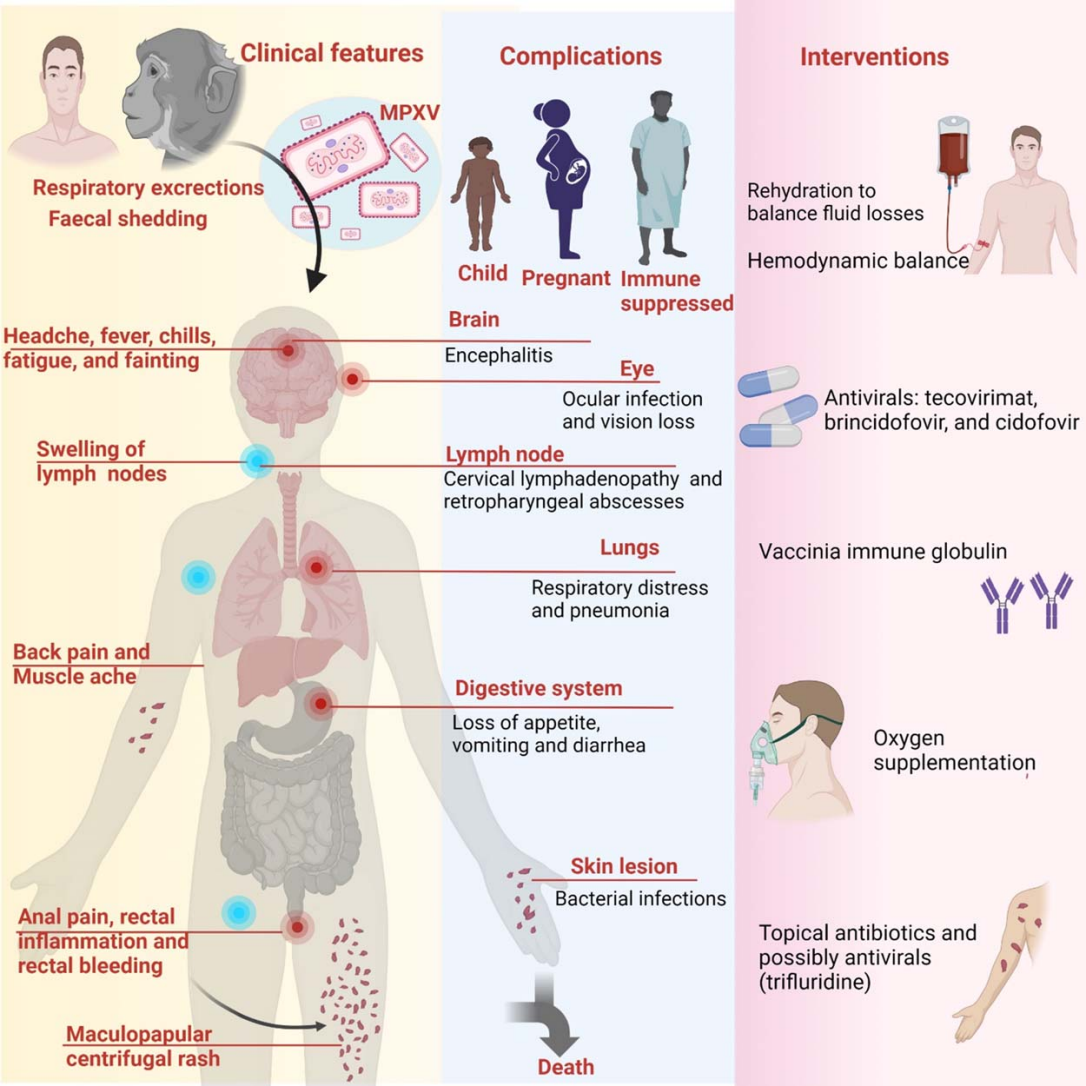
Salient clinical features and treatment options for patients with monkeypox. MPXV, monkeypox virus.

Despite continuous human-to-human transmission and a projected increase in animal-to-human transmission in a changing global epidemiological context, the international community is inadequately prepared to prevent and cure this zoonotic disease due to little research done on transmission aspects, clinical severity, and treatment choices. In addition to these difficulties, locating cases is made more difficult by the stigma and discrimination already experienced by MPX patients, and vulnerable populations are at more risk for unfavorable clinical outcomes and the spread of the disease. Although all people are susceptible to MPXV, concerns regarding stigma and discrimination linked to the present transmission of MPXV between men are at the forefront of control efforts and public discourse^21^. This should be given priority consideration since it may allow governments to persecute homosexuals if MPX is recognized as a Public Health Emergency of International Concern.

Although the MPX has been linked to an epidemic-like condition, little is known about this recently re-emerged virus. The JYNNEOS vaccine for the prevention of MPX has just been approved and recommendations have been established by the USFDA. As an added downside, there is a dearth of information from clinical trials on the currently available MPX vaccines and antivirals. Clinical trial data on currently available vaccinia vaccines, such as JYNNEOS, and antivirals like tecovirimat and brincidofovir, is lacking. The duration of immunity following the two-dose JYNNEOS vaccination series needs more investigation. There has to be clinical evidence that shows JYNNEOS and mRNA coronavirus disease-2019 vaccines are safe to use together.

The increasing number of MPX cases around the world demonstrates the urgent need to strengthen research for understanding different aspects of the virus in a better way, revamp diagnostic facilities, develop MPXV-specific effective vaccines, drugs, and therapies, and divert more resources and funds to tackle this global public health emergency. Further, our understanding of the host immune response and pathogen replication will enable us to develop newer disease prevention and treatment strategies. However, the expansion of the testing infrastructure would play an important role in preparedness for a feasible pandemic, even though MPXV’s spread is currently limited and may eventually subside. Additionally, large-scale studies are also needed to identify the specific small mammal reservoir that hosts the virus, explore the zoonotic aspects as well as the recently reported reverse zoonosis associated with MPXV, and implement needful one health approach to counteract MPXV at animal–human interface and limit such transmission^22^.

## Ethical approval

All data presented in the study has been collected from open-source platforms with proper citation and/or from media sources.

## Sources of funding

This research did not receive any specific grant from funding agencies in the public, commercial, or not-for-profit sectors.

## Author contribution

S.K.A. and K.D.: conceived and designed this paper. S.K.A., R.G.A.E., S.O.A., A.J.A., N.A.E., D.C., A.D., T.B.E., and K.D.: wrote the manuscript. S.K.A., R.G.A.E., S.O.A., A.J.A., N.A.E., D.C., A.D., T.B.E., and K.D.: revised the manuscript. The author(s) read and approved the final manuscript.

## Conflicts of interest disclosure

The authors declare that they have no known competing financial interests or personal relationships that could have appeared to influence the work reported in this paper.

## Research registration unique identifying number (UIN)

None.

## Guarantor

Sirwan Khalid Ahmed. E-mail: sirwan.ahmed1989@gmail.com

## Provenance and peer review

Not commissioned, internally peer-reviewed.

## References

[1] Centers for Disease Control and Prevention, 2022 Monkeypox Outbreak Global Map. CDC. 2022. Accessed October 5, 2022. https://www.cdc.gov/poxvirus/monkeypox/response/2022/world-map.html

[2] Ahmed SK Rashad EAA Mohamed MG . The global human monkeypox outbreak in 2022: an overview. Int J Surg 2022;104:106794. 35918003 10.1016/j.ijsu.2022.106794 PMC9624120

[3] Saijo M Ami Y Suzaki Y . Virulence and pathophysiology of the Congo Basin and West African strains of monkeypox virus in non-human primates. J Gen Virol 2009;90:2266–71. 19474247 10.1099/vir.0.010207-0

[4] McCollum AM Damon IK . Human monkeypox. Clin Infect Dis 2014;58:260–7. 24158414 10.1093/cid/cit703 PMC5895105

[5] European Centre for Disease Prevention and Control, Factsheet for health professionals on monkeypox. 2022. Accessed October 5, 2022. https://www.ecdc.europa.eu/en/all-topics-z/monkeypox/factsheet-health-professionals

[6] WHO, Multi-country monkeypox outbreak: situation update. 2022. Accessed October 5, 2022. https://www.who.int/emergencies/disease-outbreak-news/item/2022-DON390

[7] Kumar N Acharya A Gendelman HE . The 2022 outbreak and the pathobiology of the monkeypox virus. J Autoimmun 2022;131:102855. 35760647 10.1016/j.jaut.2022.102855 PMC9534147

[8] Chandran D Dhama KD Muhammad Aslam MA . Monkeypox: an update on current knowledge and research advances. J Exp Biol Agric Sci 2022;10:10679–688.

[9] Brown K Leggat PA . Human monkeypox: current state of knowledge and implications for the future. Trop Med Infect Dis 2016;1:8. 30270859 10.3390/tropicalmed1010008 PMC6082047

[10] Reynolds MG Yorita KL Kuehnert MJ . Clinical manifestations of human monkeypox influenced by route of infection. J Infect Dis 2006;194:773–80. 16941343 10.1086/505880

[11] WHO, Clinical management and infection prevention and control for monkeypox: interim rapid response guidance. 2022. Accessed October 5, 2022. https://www.who.int/publications/i/item/WHO-MPX-Clinical-and-IPC-2022.1

[12] Chakraborty S Chandran D Mohapatra RK . Clinical management, antiviral drugs and immunotherapeutics for treating monkeypox. An update on current knowledge and futuristic prospects. Int J Surg 2022;105:106847. 35995352 10.1016/j.ijsu.2022.106847 PMC9533875

[13] Rizk JG Lippi G Henry BM . Prevention and treatment of monkeypox. Drugs 2022;82:957–63. 35763248 10.1007/s40265-022-01742-y PMC9244487

[14] Chakraborty S Mohapatra RK Chandran D . Monkeypox vaccines and vaccination strategies: current knowledge and advances. An update – correspondence. Int J Surg 2022;105:106869. 36049620 10.1016/j.ijsu.2022.106869 PMC9533893

[15] Rodríguez-Cuadrado FJ Pinto-Pulido EL Fernández-Parrado M . FR – potenciales tratamientos en viruela símica (monkeypox). Actas Dermosifiliogr 2022. 10.1016/j.ad.2022.06.013

[16] Ahmed SK M-Amin HI Abdulqadir SO . Timely mental health care for the 2022 novel monkeypox outbreak is urgently needed. Ann Med Surg 2022;82:104579. 10.1016/j.amsu.2022.104579 PMC9450467 36092856

[17] Ahmed SK Saied AA Ravi RK . The 2022 monkeypox outbreak and associated psychiatric morbidities – correspondence. Int J Surg 2022;106:106913. 36113838 10.1016/j.ijsu.2022.106913 PMC9533825

[18] Ahmed SK Abdulqadir SO Hussein SH . The impact of monkeypox outbreak on mental health and counteracting strategies: a call to action. Int J Surg 2022;106:106943. 36155257 10.1016/j.ijsu.2022.106943 PMC9533932

[19] O’Laughlin K Tobolowsky FA Elmor R . Clinical use of tecovirimat (Tpoxx) for treatment of monkeypox under an Investigational New Drug Protocol – United States, May–August 2022. MMWR Morb Mortal Wkly Rep 2022;71:1190–5. 36107794 10.15585/mmwr.mm7137e1 PMC9484807

[20] Keckler MS Salzer JS Patel N . IMVAMUNE® and ACAM2000® provide different protection against disease when administered postexposure in an intranasal monkeypox challenge prairie dog model. Vaccines 2020;8:396. 32698399 10.3390/vaccines8030396 PMC7565152

[21] Human Dignity Trust, Map of Countries that Criminalise LGBT People. 2022. Accessed October 5, 2022. https://www.humandignitytrust.org/lgbt-the-law/map-of-criminalisation/

[22] Sah R Hada V Mohanty A . Recent first report of human-to-dog transmission of Monkeypox virus emphasizes an urgent need of enhancing surveillance and strengthen further explorative research to reveal its real magnitude of reverse zoonosis from other animals including pets as like. Int J Surg 2022;106:106949. 36174830 10.1016/j.ijsu.2022.106949 PMC9534089

